# Effects of Nordic walking training on quality of life, balance and functional mobility in elderly: A randomized clinical trial

**DOI:** 10.1371/journal.pone.0211472

**Published:** 2019-01-30

**Authors:** Natalia Andrea Gomeñuka, Henrique Bianchi Oliveira, Edson Soares Silva, Rochelle Rocha Costa, Ana Carolina Kanitz, Giane Veiga Liedtke, Felipe Barreto Schuch, Leonardo A. Peyré-Tartaruga

**Affiliations:** 1 Exercise Research Laboratory, Escola de Educação Física, Fisioterapia e Dança, Universidade Federal do Rio Grande do Sul, Porto Alegre, Rio Grande do Sul, Brazil; 2 Catholic University of Misiones–UCAMI–Posadas, Misiones, Argentina; 3 Centro de Educação Física e Desportos, Universidade Federal de Santa Maria, Santa Maria, Rio Grande do Sul, Brazil; Hvidovre Hospital, DENMARK

## Abstract

**Purpose:**

There is physiological and biomechanical evidence suggesting a possible advantage of using poles in walking training programs. The purpose of this proof-of-concept study was to test the hypothesis that untrained elderly training Nordic walking for eight weeks will show higher improvements on the functional mobility, quality of life and postural balance than that training without poles; more likely to occur in self-selected walking speed (primary outcome), and the locomotor rehabilitation index than the quality of life, the static balance and the dynamic stability. It was a two-arm randomized sample- and load-controlled study.

**Methods:**

Thirty-three untrained older people were randomly assigned into Nordic walking (n = 16, age: 64.6±4.1 years old) and free walking (n = 17, age: 68.6±3.9 years old) training groups.

**Results:**

Improvements in the self-selected walking speed (primary outcome, *p* = 0.011, ES = 0.42 95%CI -0.31 to 1.16), locomotor rehabilitation index (*p* = 0.013, ES = 0.36; (95%CI -0.39 to 1.10), quality of life (*p*<0.05), static balance (*p*<0.05) and dynamic variability (*p*<0.05) were found in both groups.

**Conclusions:**

The hypothesis was not supported, our findings indicated that after 8 weeks, the Nordic walking training did not result in greater improvements than free walking training for the primary outcome (self-selected walking speed) and most of the secondary outcomes (including locomotor rehabilitation index, static balance, dynamic stability, and psychological and social participation domains of quality of life).

**Trial registration:**

ClinicalTrials.gov NCT03096964.

## Introduction

Physical activity programs for health promotion in elderly are being implemented, such as intervention with aerobic, resistance and flexibility exercises. Consequently, it seems to be essential to know how effective the physical activity interventions are comparatively. As the life expectancy in the worldwide population continues to rise, there is an increasing interest in maintaining and improving the physical activity levels and function in older adults [1–4].

The Nordic walking (NW) is a relatively new modality of walking training that is performed using ergonomically well-designed poles. The main purpose of the use of the poles is the recruitment of upper limbs muscles to produce ground reaction forces resulting in an increased energetic expenditure. Therefore, the NW is considered a physical activity recommended for the elderly [3–5]. It has been reported an increasing amount of studies on gains in physical conditioning, functional fitness, balance, strength, walking speed, and quality of life after NW training in untrained older people [3–9].

However, there have been considerable inconsistencies in the training methods applied in these studies. Some randomized clinical trials evaluated the effect of the NW in the elderly (lasting between 6 and 12 weeks) by training only at SWS, without supervision by NW instructors [6]. And other studies used low-intensity exercises (60% of predicted maximum heart rate) [7] or controlled the intensity of the training only by the rating of perceived exertion [3], and others did not control the volume and intensity of the sessions [5,9]. In one unique study, NW instructors monitored the heart rate, and rating of perceived exertion [4], however, exercise intensities were lower (100–120 bpm) than those used in the present study. Differently from the previous studies, our study detailed load control of each session in basis of volume (session time) and intensity (controlled by cardiac monitor and following the periodization of the training according to the percentage of the ventilatory threshold) in both intervention groups (NW and FW). Still, all sessions were attended by at least three trained NW instructors who controlled the NW technique and the intensity of the exercises of each participant, ensuring full control of the intervention performed.

Thus, the purpose of the present randomized clinical trial was to assess the effects of eight weeks of NW training and FW training on SWS, locomotor rehabilitation index (LRI), quality of life, static balance and dynamic variability of untrained elderly people. We hypothesized that untrained elderly training NW for eight weeks will show higher improvements in quality of life, static balance, dynamic variability, SWS and LRI than that training FW due to the more complex control required by NW [10,11].

## Materials and methods

### Experimental design

**Fig 1:**
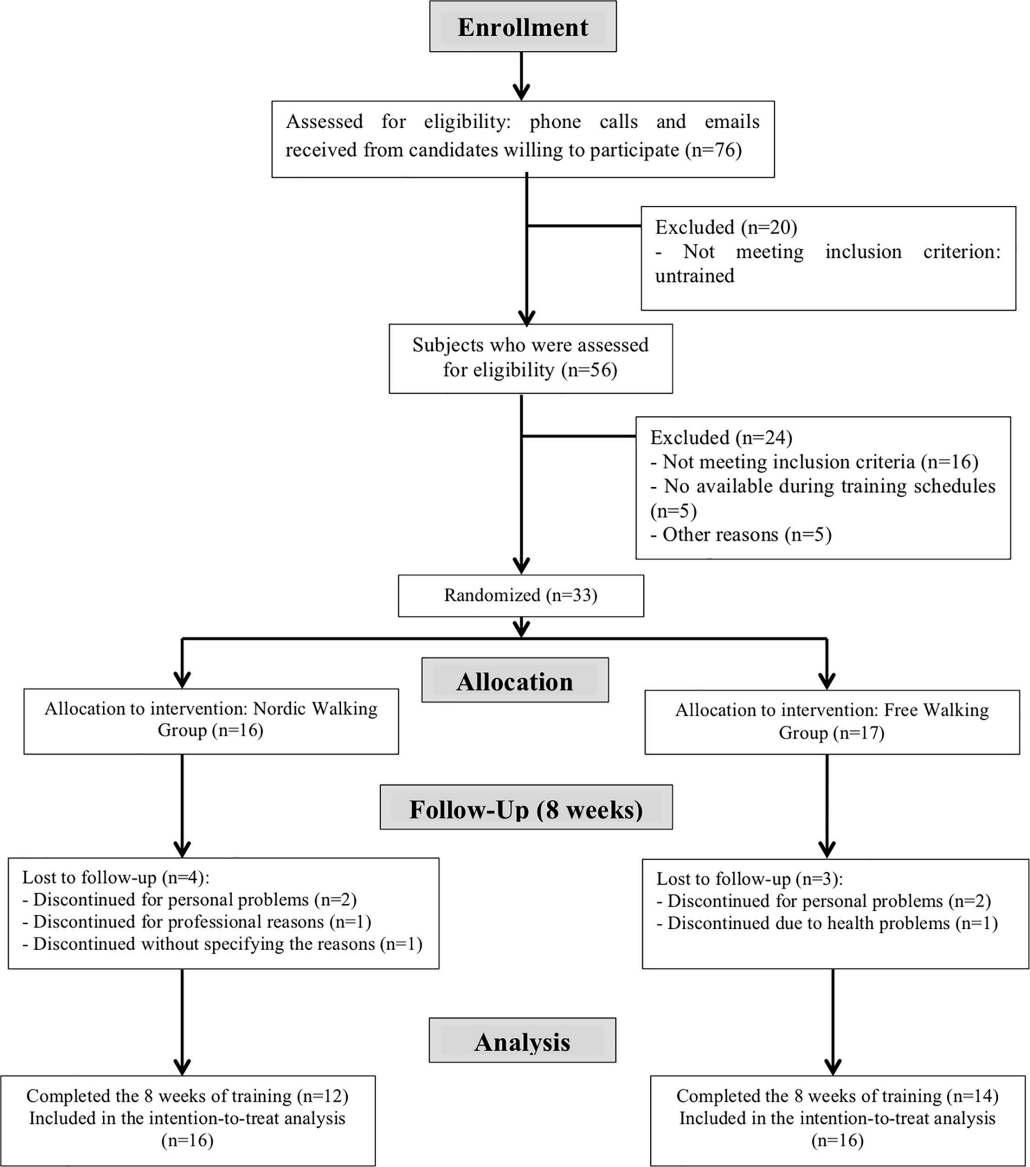
Flowchart of the study participants.

The study is a randomized controlled clinical trial in parallel, designed in accordance with the CONSORT 2010 guidelines (S1 File) [12]. The flowchart of the study was reported in Fig 1. This trial has been registered in Clinical Trials (NTC03096964, S2 File). This study was registered after starting the recruitment because our institution (ESEFiD-UFRGS) only authorizes the registration after having evaluated the project in the University's Qualifying Exam and after approval of the local ethics committee (in November to 2014, S3 File and S4 File). And, only at this point, we were authorized to do the register in the Clinical Trials. The authors confirm that all on-going and related studies for this intervention were recorded. There were no alterations in the methods after beginning of the study and randomization. All evaluations and training sessions were conducted in the School of Physical Education, Physical Therapy and Dance of the Universidade Federal do Rio Grande do Sul (UFRGS) at Porto Alegre–Brazil. The familiarization period was in August 2015, and the pre-training data collect in September 2015. Training period (October/November) and post-training data collect (December) took place in 2015. All participants read and signed a free and informed consent form before starting their participation in the study that was previously approved by the university research ethics committee (Number 878.736 in November 2014).

To investigate the effects of eight weeks of NW training and FW training on SWS, LRI, quality of life, static balance and dynamic variability of untrained elderly people, both groups (NW and FW) were trained accordingly and were evaluated before and after the training period. The same assessors who were blinded to the training groups conducted the test sessions, and the same equipment was used in all of the sessions. The participants were instructed to maintain their eating habits during the study period. Here we are reporting SWS, LRI, quality of life, static balance and dynamic variability, parameters of pendular mechanism (external, internal, and total mechanical work, recovery, cost of transport), electromyographic parameters (average signal and co-contraction of the anterior deltoid, triceps brachii, vastus lateralis, biceps femoris, tibialis anterior, and gastrocnemius medial muscles, heart rate, and the rate of perceived exertion. In a subsequent paper, we will report parameters of pendular mechanism (external, internal, and total mechanical work, recovery, cost of transport), electromyographic parameters (average signal and co-contraction of the anterior deltoid, triceps brachii, vastus lateralis, biceps femoris, tibialis anterior, and gastrocnemius medial muscles, heart rate, and the rate of perceived exertion. Then, the outcomes parameters of pendular mechanism (external, internal, and total mechanical work, recovery, cost of transport), electromyographic parameters (average signal and co-contraction of the anterior deltoid, triceps brachii, vastus lateralis, biceps femoris, tibialis anterior, and gastrocnemius medial muscles, heart rate, and the rate of perceived exertion are not considered as unreported, and they are removed from the denominator.

### Participants

Thirty-three sedentary elderly people were randomized into two groups. The experimental group performed walking with poles training (NW, n = 16 participants) over 8 weeks. While the control group (FW, n = 17 participants) did the same intervention but walking without poles training during the same period. The following inclusion criteria were applied: 1) aged between 60 and 80 years old; 2) being untrained, understood as those older people who were at least six months without practicing structured or systematized physical activities; 3) non-smoking; 4) do not present chronic pain, migraine or nausea in daily life, or history of labyrinthitis, or any other health condition that could prevent or limit the realization of the sessions or assessments. The baseline characteristics of the sample are shown in Table 1.

**Table 1:** Sample characterization. Mean and confidence interval (95% CI) for age, height, body mass, body mass index, waist/height ratio (WAI/HEI), sum of skinfolds, lower limb length (LLL), and medication.

Variable	FW group (n = 16)	NW group (n = 17)
	Mean (SD)	Mean (SD)
Age (years)	68 (3.9)	64 (4.1)
Height (cm)	162 (10.3)	165 (7.5)
Men, n (%)	4 (44.4)	5 (55.6)
Body Mass (kg)	74 (14.5)	81 (10.0)
Fat Percentage (%)	31 (27 to 35)	32 (27 to 37)
Body Mass Index (kg.m^-2^)	28 (4.7)	29 (3.9)
WAI/HEI	0.87 (0.08)	0.91 (0.07)
Sum of Skinfolds (mm)	132 (18.4)	132 (28.7)
LLL_right_ (cm)	83 (5.7)	85 (5.0)
LLL_left_ (cm)	83 (5.4)	86 (5.5)
Medication (n medicated individuals)		
Hypoglycaemic (%)	3 (19)	4 (24)
Anti- hypertensive (%)	5 (31)	7 (41)
Hypolipidemic (%)	8 (50)	7 (41)
Diuretics (%)	3 (19)	1 (6)
Hypothyroidism (%)	1 (6)	3 (18)
Humour (%)	4 (25)	4 (24)

After randomization, there were no alterations in the methods after the beginning of the study. All evaluations and training sessions were conducted in the School of Physical Education, Physical Therapy and Dance of the Federal University of Rio Grande do Sul (UFRGS) of Porto Alegre–Brazil. All participants read and signed a free and informed consent form before starting their participation. This study was conducted according to the Helsinki Declaration and was approved by the Ethics Committee of the Federal University of Rio Grande do Sul, Brazil under registration no. 878.736. The flow chart of the study was reported in results.

### Procedures

#### Physical assessment

Participants performed one month of familiarization with NW technique (one weekly session of 45 minutes). After familiarization, participants attended the laboratory to undergo the pre-training period assessments.

On the first visit (day 1) anthropometric; quality of life (WHOQOL-OLD and WHOQOL-BREF) and static balance in force platform assessments were performed. On the second visit (day 2), participants performed an incremental test on the treadmill. Moreover, in the third visit (day 3) to the laboratory, participants performed the evaluation of SWS and walking at submaximal constant speeds (1, 2, 3, 4 and 5 km.h^-1^) on the treadmill, where SWS, LRI, and dynamic stability were assessed in the pre-training moment. The sessions were performed with a minimum interval of 48 hours. After the conclusion of these assessments, the participants were randomized into NW and FW groups and performed eight weeks of training. Afterward the training period, the participants went back to the laboratory to undergo the same evaluations corresponding to post-training moment.

#### Familiarization with treadmill and Borg’s scale

Participants were informed about security mechanisms and walked on the treadmill at different speeds (with a gradual increase of 0.5 km.h^-1^). Thus familiarization lasted approximately 15 minutes. In this occasion, they were also familiarized with Borg’s Scale of 1 to 10 [13,14], indicating the rating of perceived exertion during the intervals of the walking tests and with neoprene mask that would be used for the collection of maximal oxygen consumption, and the walking at different submaximal speeds on treadmill.

#### Static balance test

A force platform was used (Model AMTI OR6-5, Inc., Watertown, MA) for the measurement of the forces and moments in three dimensions (F_x_, F_y_, F_z_, M_x_, M_y_ and M_z_). The evaluation was performed by an individual standing on the platform–bipedal support with bare and united feet–in the situations without blindfold (eyes open) and with blindfold (eyes closed). In both conditions the participants should keep their arms rested beside the body and, in the open eyes condition, the individuals should be stared at a point marked on the wall (in the height of the eyes about three meters away). Besides that, the participant evaluated was instructed not to move or speak during the collection of the data (should maintain orthostatic position). The order of the tests was raffle by an evaluator after initial instructions. The platform was calibrated before each collection. When the participant was properly placed in the position of the test, the data collection was started. Closed and open eyes situations were executed three times, with a duration of 30 seconds each and interval of one minute between them.

Force and moment data were exported from Nexus software, and then the center of pressure (COP) was calculated in Matlab software (The Mathworks, Natick, USA). The signals coming from the force platform had a gain of 4000 times and were collected at 2000 Hz. In the processing of raw data, a mathematics routine was used (with a Butterworth low-pass filter of 4^th^ order and with a cut-off frequency of 10 Hz defined by the residual analysis method). The postural balance was analyzed through the maximal amplitude of COP_x_ and COP_y_ displacement; the average amplitude of COP_x_ and COP_y_ displacement; average speed of COP_x_ and COP_y_ displacement, and total average speed of COP displacement of the three attempts for each situation was used (closed and open eyes).

#### Incremental test on the treadmill

Previously to the incremental test, resting systolic and diastolic blood pressure of the participants was measured. If they were with elevated blood pressure (higher than 140 mmHg and 90 mmHg for systolic and diastolic blood pressure, respectively), the test was rescheduled. Participants with normal blood pressure performed the incremental maximal effort test in the treadmill with the protocol proposed by Bruce [15]. The speed and inclination were progressively incremented until maximal effort (starting at 2 km.h^-1^ and 2% incline and increasing every 2 minutes plus 1 km.h^-1^ and 2% inclination.). The test was finished when the participants reached exhaustion and communicated it to the researchers through visual signs. Two trained researchers blinded to the allocation of the participants independently determined all the values of maximal oxygen consumption, ventilatory thresholds and heart rates in these situations. The value of oxygen consumption in the second ventilatory threshold and heart rate in the second ventilatory threshold were used to prescribe the eight weeks of training.

#### Walking test on treadmill and dynamic stability

Before the beginning of the kinematic data collection the participants were prepared by trained researchers, who positioned 35 reflective markers on anatomic points of interest according the model Plug-in-Gait Full-Body of the software Nexus 1.8.5 for the collection of Vicon Motion Capture System 3D kinematics (Oxford, United Kingdom), with six cameras of the model Bonita, with a 200 Hz sampling frequency. Calibration of the space volume of the collection was made, according to the orientations of the manufacturer’s manual before the beginning of the collection and, if necessary, the calibration procedure was repeated.

After placing the reflective markers, the participant positioned himself/herself on the treadmill (INBRAMED, model ATL-Inbrasport, Porto Alegre, Brazil) and were performed the static collection of the images. The participants performed more 5 to 10 minutes of familiarization on the treadmill, and only after that, SWS was measured.

A trained evaluator gave the instructions for the participants to determine their SWS in the treadmill. The treadmill speed indicator panel was covered, so the individual did not know at which speed he/she was walking. Thus, the researcher gave the command to the participant, who should walk at the most comfortable speed for him/herself, then representing the walking speed that he/she could sustain for a long period of time. The treadmill speed was gradually decreased or increased in 0.5 km.h^-1^ several times, until the participant was sure that that speed was his/her most comfortable walking speed, and only after this confirmation, treadmill panel was uncovered, and the SWS was registered.

Posteriorly, the participants performed different submaximal walking speeds in the treadmill during five minutes in each one of these speeds (1, 2, 3, 4, and 5 km.h^-1^) alternating with five minutes of rest between them. Kinematic data were recorded in the minute four of each walking test, with duration of 30 seconds. The oxygen consumption was registered during the intervals and the tests are re-started when the values reached the pre-test values.

**Fig 2:**
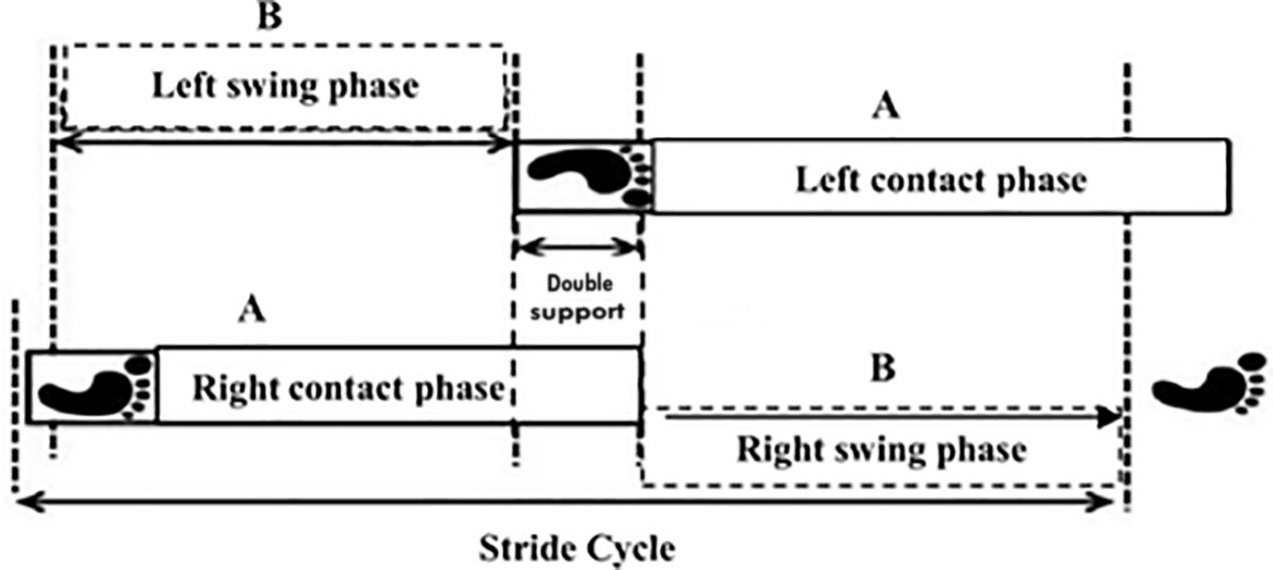
Definition of a stride cycle. The Contact Time (CT) is the time, during the stride cycle, in which the feet (right or left) is in contact with the ground (A). Swing phase time (ST) is the time, during stride cycle, in which the feet (right or left) is not in contact with the ground (B), adapted from Oliveira et al. (2013).

Ten strides were used to analyzing the kinematic data using the Nexus software, and then, were obtained the contact events (touchdown) and unload events (take off) of the foot with the ground. The touchdown was manually determined by visualizing the minimum value in the vertical displacement graph of each step of the point located in the calcaneus, whereas take off was manually determined by visualizing the first positive displacement of the base point of the third metatarsal of each step in the vertical axis. The determination of the variables corresponding to the gait cycle was performed (contact time, s; swing time, s; and stride frequency, Hz; Fig 2), from the transformation of the quantity of frames of each phase of the stride for time data, multiplying the quantity of frames by *dt (dt = 1/f)*, where *f* is sampling frequency of the images acquisition. For the obtainment of stride length (m), we used the equation stride length = speed/stride frequency.

As an indication of dynamic stability, we obtained the gait variability (stride-to-stride fluctuations) determining the coefficient of variation for the contact time, stride time, stride length, and stride frequency (CoV_CT_, CoV_ST_, CoV_SL_, CoV_SF_, respectively). Specifically, at every ten cycles, we divided the standard deviation of each variable by its respective mean values multiplied by 100, using the following equation *CoV* = 100.(*standard deviation/mean*) [16–18].

Regarding the use of the locomotor rehabilitation index (LRI), which is a method that allows the determination of how close SWS is in comparison to the optimal walking speed, we calculated the theoretical optimal walking speed of the elderly (*OPT*_*T*_), using a mathematical model as described in Eq 1 [19]:

$${OPT}_{T}=\sqrt{0.25.9.81.\;lower\;limb\;length}$$ Eq 1

The *OPT*_*T*_ is the absolute speed at which participants spend less metabolic energy per meter of traveled distance, 0.25 represents the Froude Number in which many animals spend less metabolic energy to walk, 9.81 is the gravitational acceleration, and the lower limb length is in meters. After that, to quantify the LRI, it is needed to know SWS and theoretical optimal walking speed. The ratio between SWS and theoretical optimal walking speed multiplied by 100 indicates LRI, according to Eq 2:

$$LRI=100.\frac{SWS}{{OPT}_{T}}$$ Eq 2

Therefore, when LRI value is closer to 100, it indicates that the participants are closer to their theoretical optimal walking speed [19,20].

### Training protocol

**Fig 3:**
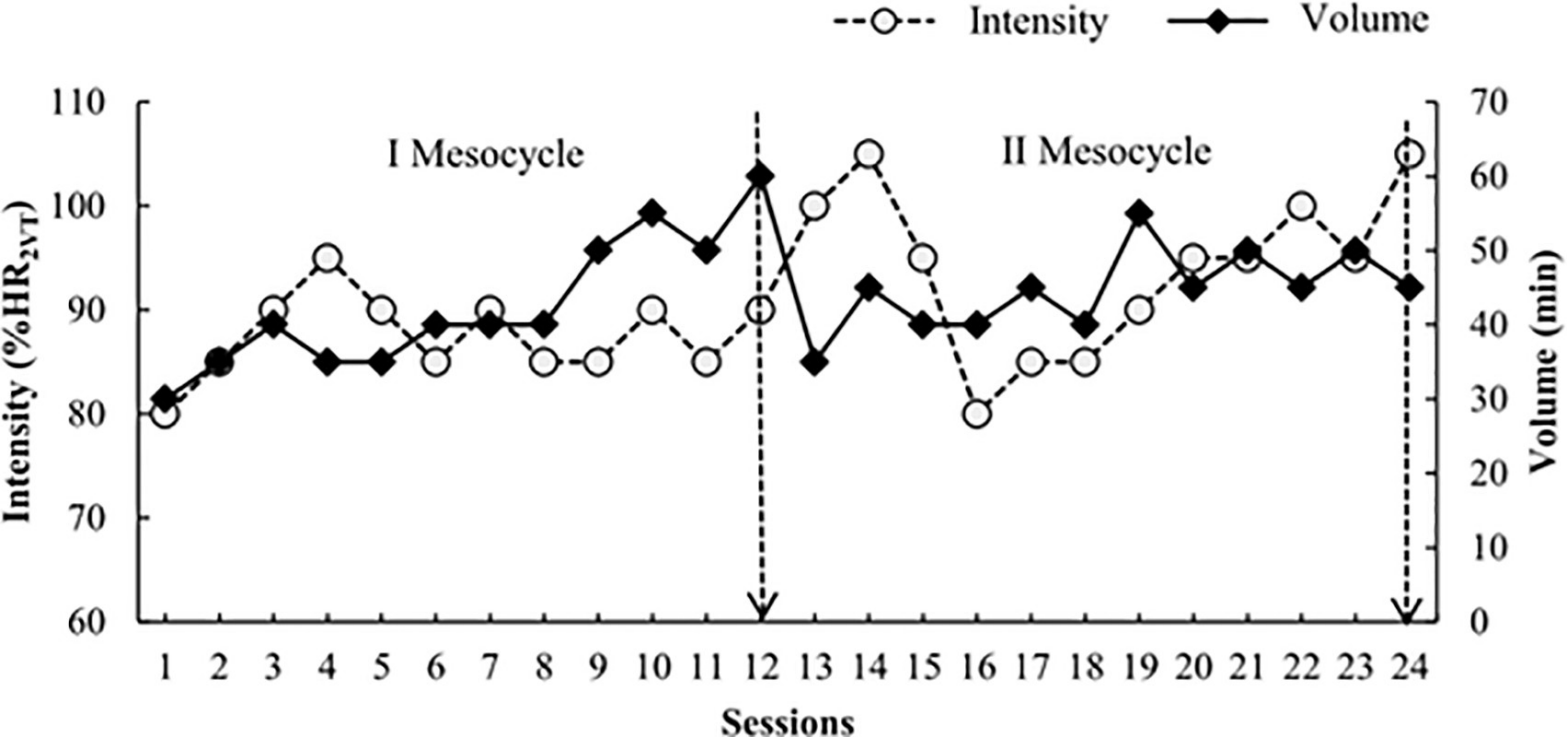
Model of training periodization. The average effort intensity (% heart rate at the second ventilatory threshold, %HR_2VT_) and training volume (in minutes) at every session during the first (I Mesocycle) and second (II Mesocycle) mesocycles.

The training period had eight weeks in duration, with three weekly sessions (24 sessions in total). The volume (session time in minutes) and intensity (percentage of heart rate at the second ventilatory threshold reached by the participants during the sessions) were equal for both groups. The heart rate was controlled by a cardiac monitor (POLAR, S610 model, Finland) during training sessions, with the groups only differing by the use (or not) of the poles during the walks. Training periodization in the different sessions is described as follows: in the warming-up, all sessions started with 5 minutes of warming-up; then followed the main part, with the time referring to the aims of volume and intensity of the session (Fig 3, Training Periodization); finally the back to calm was held, all sessions ended with five minutes of back to calm and posteriorly stretching was performed. The training protocols are shown in details in TIDieR checklist (S5 File).

### Outcomes

The SWS was considered as the primary outcome variable. The results obtained from the quality of life test, the static balance test, the LRI and the dynamic stability were used as secondary outcomes of these interventions. All data can be seen in the supplementary material (S1 Table).

### Sample size

The sample size was determined by using Gpower Software (version 3.1) adopting a power as 1-beta error probability: 95%, effect size: 0.90, and error assumed as alpha: 0.05 (two-sided) for the calculations, considering the outcomes (SWS, LRI, the static balance test). The minimal clinically important difference for the SWS have been reported as 0.1 m/s. Effect sizes ranging from 0.25 to 0.50 extracted from previous studies comparing NW and FW groups [20–26] were used. After calculation, 20 participants were indicated for allocation equally for each group, 10 participants in NW group and 10 in FW group. We decided to accrue more participants in each group, due to possible dropout. Therefore, the study’s target sample size was set at 33 (see Fig 1). The protocol study is in the supplementary material (S6 File).

### Randomization

This study was designed as a randomized controlled clinical trial in parallel, with an allocation index of 1:1. Participants were allocated to the two groups by simple randomization (binary random list, www.randomization.org). Allocation concealment was performed by a sequentially numbered list, in which a blinded evaluator indicated to each group, and each number (participant) corresponded. The researcher that conducted the allocation was not involved with the study, in order to maintain the confidentiality of the allocation and blinding of the study. Randomization and allocation processes were performed after the conclusion of the familiarization with NW technique and pre-training tests, before starting the training period.

### Blinding

All variables were acquired in the pre- and post-training data collect by trained researchers, who were blinded to group allocation and were not involved with the study or training. All participants were not blinded to intervention training (NW or FW).

### Statistical analysis

Descriptive analysis (mean ± standard error of the mean) was used to report the results. Primary and secondary outcomes were analysed in intention-to-treat analysis. We applied the Generalized Estimating Equations to test the main effects. For SWS, LRI, and quality of life outcomes, “group” and “time” were used as factors; for static balance outcomes, “group”, “time” and “situation” were used as factors; for dynamic balance outcomes, “group”, “time” and “speed” were used as factors. The outcomes were treated as normal distribution, with a connection identity function, therefore, normality and homogeneity tests were not needed. The working correlation matrix used was unstructured and robust estimator covariance matrix.

The missing data of participants who dropped out during the intervention period (post-training data) were estimated using maximum likelihood estimation by the automatic regression imputation method within the GEE analysis.

Also, effect size (by Cohen’s Method) was calculated using post-training values between NW and FW groups and were classified as small effect (between 0.2 and 0.5), moderate (between 0.5 and 0.8) and large effect (0.8 or more) [27]. These results are presented by mean and confidence interval of 95%. Significance level adopted was α = 0.05 for all tests. Statistical processing was performed by a highly trained researcher who was blinded to the participants, using SPSS software (Statistical Package for Social Sciences for Mac, version 22.0).

## Results

### Participant baseline characteristics

Sample characteristics at the beginning of the training were shown in Table 1. The participants were divided randomly to NW group with n = 16 participants, age: 64.6±4.1 years old, body mass: 81.5±10.7 kg, and height: 166.3±7.5 cm; and FW group with n = 17 participants, age: 68.6±3.9 years old, body mass: 74.6±14.5kg and height: 161.6±10.3 cm.

Seven of the 33 participants of the study dropped out during the training period [three participants of FW group (two for personal reasons and other because of health issue) and four participants of NW (two for personal reasons, one because of health issue, and one did not state the reason)] representing 18.75% of sample loss. In this way, 26 participants finished the intervention and completed the assessments (NW = 12 and FW = 14). Participants who completed the intervention had a minimum frequency of 90%. It is important to highlight that the post-training missing data of these seven participants who dropped out during the interventions were imputed (one data for each outcome, per participant), and the data of all randomized participants (33 patients) were included in the statistical analysis. There is no missing data for the remaining participants.

**Fig 4:**
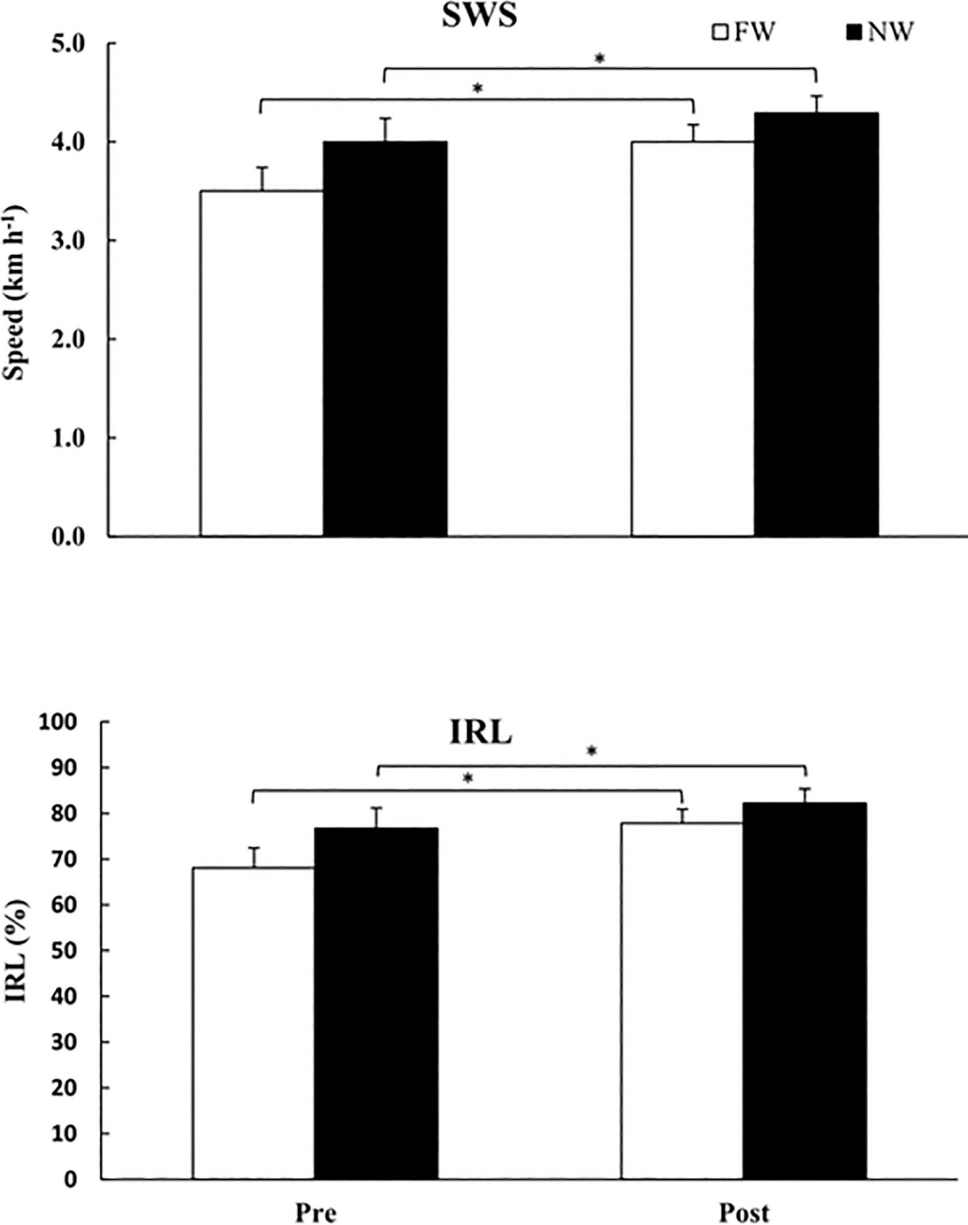
Results of the self-selected walking speed (SWS, top panel) and the locomotor rehabilitation index (LRI, bottom panel). Free walking group (FW, white column) and Nordic walking group (NW, black column) in pre- and post-training moments. Mean and standard errors. * Symbols represent significant differences in time factor (*p* = 0.011 and *p* = 0.013, respectively).

The intention-to-treat analysis of the primary outcome the SWS, demonstrated that SWS had influence only in factor time (*p* = 0.003), where it was determined that there was an increase of SWS in both groups, FW in pre-training moment from 3.50 km.h^-1^ to post-training of 4.00 km.h^-1^, and in NW from 4.00 km.h^-1^ in pre-training to 4.29 km.h^-1^in post-training (Fig 4, top panel). There were no differences in group factor (*p* = 0.270).

LRI showed significant increments only in factor time (*p* = 0.015) for both groups, whereas FW increased from 68.08% in pre-training to 77.85% in post-training; and NW group from 76.73% to 82.26% in the post-training moment (Fig 4, bottom panel). There were no differences in factor group (*p* = 0.132).

The intention-to-treat analysis of quality of life (Table 2) revealed significant time effects in the psychological domain in WHOQOL-BREF (*p* = 0.014) and social participation of WHOQOL-OLD (*p*<0.001). There were significant group effects in the environment (*p* = 0.016), and social relationships domains of WHOQOL-BREF (*p* = 0.030) were also found.

**Table 2:** Results from GEE model effects tests for quality of life test variables of WHOQOL-BREF and WHOQOL-OLD in the different domains. * Symbol represents significant differences in time factor (pre and post-training), and different capital letters represent significant differences in group factor (FW and NW).

		Pre-training	Post-training	*p*–value	
Quality of life	Intervention	Mean ± SE	Mean ± SE	Group	Time
BREF—general	FW	65.38 **±**4.11	67.85 **±**4.32	0.092	0.674
	NW	72.32 **±**5.38	77.67 **±**3.61		
BREF–Physical	FW	60.39 **±**4.57	64.56 **±**4.84	0.270	0.051
	NW	65.05 **±**4.36	72.66 **±**3.45		
BREF–Psychological	FW	66.02 **±**4.52^*^	71.42 **±**4.64^*^	0.907	**0.014**
	NW	63.98 **±**4.91^*^	74.79 **±**2.26^*^		
BREF–Environment	FW	61.29 **±**3.39^**A**^	60.65 **±**4.36^**A**^	**0.016**	0.645
	NW	73.66 **±**3.75^**B**^	73.40 **±**3.92^**B**^		
BREF–Social relationship	FW	62.82 **±**3.58^**A**^	65.61 **±**5.61^**A**^	**0.030**	0.649
	NW	70.83 **±**4.27^**B**^	73.37 **±**3.37^**B**^		
OLD–Total	FW	65.62**±**3.63	70.65 **±**3.93	0.300	0.596
	NW	72.75 **±**3.47	71.30 **±**2.43		
OLD–Social participation	FW	60.09 **±**5.41*	77.40 **±**5.02*	0.413	**<0.001**
	NW	70.41 **±**4.30*	77.08 **±**3.55*		
OLD–Sensory abilities	FW	75.00 **±**2.94	70.53 **±**4.78	0.260	0.139
	NW	78.75 **±**2.75	76.04 **±**5.09		
OLD–Past, present and future activities	FW	68.22 **±**6.22	72.76 **±**4.57	0.601	0.495
	NW	74.58 **±**3.19	73.43 **±**4.04		
OLD–Intimacy	FW	64.28 **±**7.18	65.62 **±**7.16	0.393	0.219
	NW	76.52 **±**5.17	66.66 **±**8.63		
OLD–Death and dying	FW	63.83 **±**6.41	62.50 **±**5.54	0.564	0.963
	NW	64.16 **±**5.57	68.18**±**4.13		
OLD–Autonomy	FW	66.34 **±**3.63	64.42 **±**4.87	0.369	0.396
	NW	70.00 **±**3.18	72.91 **±**3.48		

All static balance variables (ie: Maximum Amplitude of COP_X_ and COP_Y_, Average Amplitude COP_X_ and COP_Y_, Average Speed COP_X_ and COP_Y_, and Average Speed COP_TOTAL_) were modified by the open eyes versus eyes closed situation (*p*<0.001), presenting lower values in the open eyes when compared to the closed eyes situation for both groups and moments (Table 3). The average speed of the COP was modified by the time factor, with CoP_X_ Average Speed (*p* = 0.004), CoP_Y_ Average Speed (*p* = 0.031) and COP_TOTAL_ Average Speed (*p* = 0.009) presenting lower values at the post-training moment when compared to the pre-training moment in both groups and situations. The results of the static balance evaluated by force platform in open and closed eyes conditions can be observed in Table 3.

**Table 3:** Mean, SE, and differences from GEE model effects tests for static balance variables in force platform. * symbol represents differences between with the closed and open eyes situations in inter-group relation (within each group). Different capital letters represent significant differences of time factor (pre and post-training).

Variable	Group/ Situation	Pre Mean± SE	Post Mean± SE	Group	Time	Situation (BL/WB)
				*p*		
**AM**_**ax**_ **COP**_**X**_ **(mm)**	FW_BL_	28.11**±**1.68*	27.49**±**1.21^*^	0.456	0.216	**<0.001**
	FW_WB_	24.41**±**1.42	27.84**±**3.54			
	NW_BL_	31.00**±**1.19^*^	32.01**±**2.33^*^			
	NW_WB_	25.10**±**1.24	26.09**±**1.48			
**AM**_**ax**_ **COP**_**Y**_ **(mm)**	FW_BL_	36.25**±**2.66^*^	34.75**±**2.92^*^	0.162	0.227	**<0.001**
	FW_WB_	29.46**±**1.71	32.10**±**3.08			
	NW_BL_	30.56**±**1.78^*^	33.32**±**1.96^*^			
	NW_WB_	26.65**±**1.66	28.16**±**1.21			
**A**_**verage**_**A** **COP**_**X**_ **(mm)**	FW_BL_	4.21**±**0.26*	4.50**±**0.17^*^	0.079	0.499	**<0.001**
	FW_WB_	4.00**±**0.30	3.81**±**0.30			
	NW_BL_	4.77**±**0.20^*^	5.02**±**0.36*			
	NW_WB_	4.00**±**0.22	4.42**±**0.23			
**A**_**verage**_**A** **COP**_**Y**_ **(mm)**	FW_BL_	5.52**±**0.37^*^	5.33 **±**0.53^*^	0.243	0.540	**0.001**
	FW_WB_	4.56**±**0.32	4.61**±**0.38			
	NW_BL_	4.55**±**0.25*	4.98**±**0.29^*^			
	NW_WB_	4.26 **±**0.30	4.46**±**0.21			
**A**_**verage**_ **S**_**peed**_ **COP**_**X**_ **(mm/s)**	FW_BL_	14.77**±**1.6^*A^	12.47±1.66^*B^	0.811	**0.004**	**<0.001**
	FW_WB_	10.55**±**0.73^**A**^	9.42**±**0.90^**B**^			
	NW_BL_	14.68**±**1.15^*A^	13.52**±**1.17^*B^			
	NW_WB_	10.82**±**0.70^**A**^	9.52**±**0.73^**B**^			
**A**_**verage**_ **S**_**peed**_ **COP**_**Y**_ **(mm/s)**	FW_BL_	19.03**±**1.92^*A^	16.08**±**1.4^*B^	0.571	**0.031**	**<0.001**
	FW_WB_	13.62**±**1.13^**A**^	11.19**±**0.87^**B**^			
	NW_BL_	16.69**±**1.68^*A^	16.40±1.39^*B^			
	NW_WB_	11.72 **±**0.83^**A**^	11.65 **±**0.88^**B**^			
**A**_**verage**_ _**Total**_**S**_**peed**_ **COP** **(mm/s)**	FW_BL_	26.72**±**2.69^*A^	22.54**±**1.90^*B^	0.807	**0.009**	**<0.001**
	FW_WB_	19.07**±**1.39^**A**^	16.25**±**1.32^**B**^			
	NW_BL_	24.74**±**2.03^*A^	23.55**±**1.88^*B^			
	NW_WB_	17.64**±**1.03^**A**^	16.71 **±**1.15^**B**^			

Note: abbreviations of the different situations of body static balance, for AM_ax_ COP_X_ (mm) and AM_ax_ COP_Y_ (mm) represent the maximal amplitude of COPx and COPy displacement, respectively. For A_verage_A COP_X_ (mm), A_verage_A COP_Y_ (mm) represent the average amplitude of COPx and COPy displacement; respectively. Average Speed COP_X_ (mm/s) and Average Speed COP_Y_ (mm/s) are the average speed of COPx and COPy displacement, and Average total speed of COP displacement (COPx plus COPy) of the three attempts for each situation was used (WB- open eyes; and BL- eyes closed).

**Fig 5:**
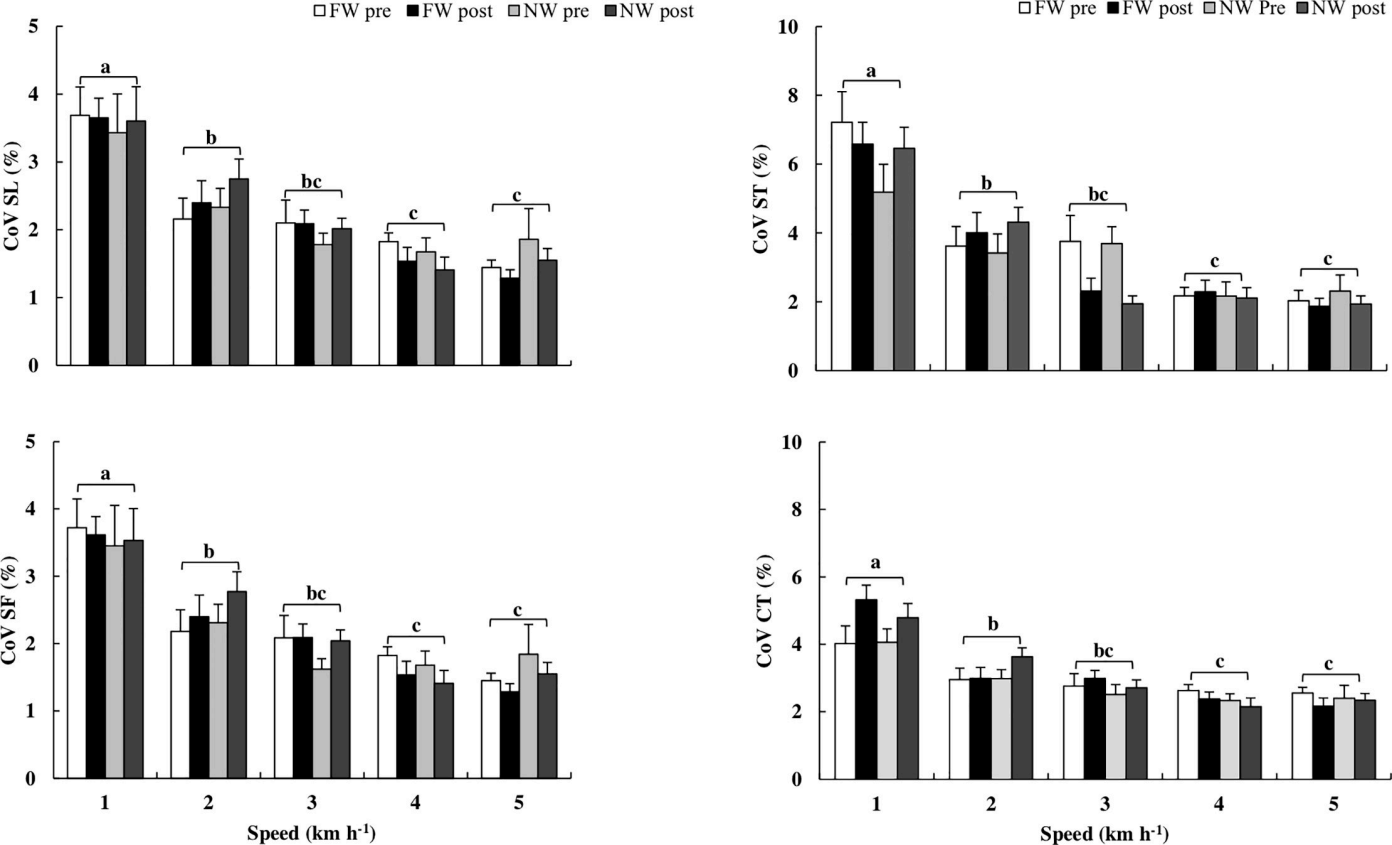
Results of gait variability through the coefficient of variation (CoV) of spatiotemporal parameters. Left side, CoV of stride length (CoV_SL_, top panel) and CoV of stride frequency (CoV_SF_, bottom panel), and, in the right side, the CoV of stride time (CoV_ST_, top panel), and CoV of contact time (CoV_CT_, bottom panel). FW group results of pre- and post-training moments are observed in white and black columns, respectively. NW group results of pre- and post-training moments are observed in light grey and dark grey, respectively. Data presented in mean and SE. Different letters represent significant differences (p<0.001) between speeds.

Statistical results of dynamic stability (Fig 5. Left Panel) regarding to coefficient of variation (CoV) of stride length (CoV_SL_), demonstrate that it decrease with speed factor (*p*<0.001). There were no differences in the group factor (*p* = 0.929). The CoV of stride frequency (CoV_SF_), was decreased as a function of the speed factor (*p*<0.001). This variable was not affected by group factor (*p* = 0.991) and by time factor (*p* = 0.973). Values are observed in Fig 5. Left Panel.

Still, in relation to dynamic stability results (Fig 5. Right Panel) corresponding to CoV of swing time (CoV_ST_), these results demonstrated that they were also affected by walking speed (*p*<0.001), without significant influence of group factor (*p* = 0.442), and time factor (*p* = 0.432). Regarding CoV of contact time (CoV_CT_), it was observed that CoV_CT_ was decreased with speed (*p*<0.001). The other factors did not affect CoV_CT_, as group factor (*p* = 0.694), time factor (*p* = 0.148). Results of both variables in different walking speed can be observed in Fig 5. Right Panel.

The effect sizes of SWS, LRI, quality of life test, and static balance parameters, are in Table 4 (all effect sizes are listed in supplementary material, S2 Table).

**Table 4:** Effect size results of post-intervention comparisons of NW and FW groups (left column). Values in mean and lower and upper limits of 95% Confidence Interval, for the SWS, LRI, quality of life questionnaire and static balance parameters. Comparison of means between groups is in the right column.

Variables:	Mean (95% CI)	Comparison
**SWS**	0.42 (-0.31 to 1.16)	NW > FW
**LRI**	0.36 (-0.39 to 1.10)	FW > NW
BREF–Physical	0.50 (-0.28 to 1.29)	NW > FW
BREF–Psychological	0.25 (-0.53 to 1.02)	NW > FW
BREF- Environment	0.79 (-0.01 to 1.59)	NW > FW
BREF–Social Relation	0.90 (0.09 to 1.71)	NW > FW
BREF–General	0.64 (-0.15 to 1.43)	NW > FW
OLD–Total	0.05 (-0.72 to 0.82)	NW > FW
OLD–Social Participation	0.02 (-0.75 to 0.79)	FW > NW
OLD–Sensory abilities	0.28 (-0.49 to 1.06)	NW > FW
OLD–Past, present and future activities	0.04 (-0.73 to 0.81)	NW > FW
OLD–Intimacy	0.03 (-0.74 to 0.80)	NW > FW
OLD–Death and dying	0.30 (-0.47 to 1.08)	NW > FW
OLD–Autonomy	0.53 (-0.26 to 1.31)	NW > FW
**- Balance parameters** **Static:**		
AMax COP_X_ closed eyes	0.60 (-0.16 to 1.36)	FW > NW
AMax COP_X_ open eyes	0.16 (-0.58 to 0.90)	NW > FW
AMax COP_Y_ closed eyes	0.14 (-0.60 to 0.88)	NW > FW
AMax COP_Y_ open eyes	0.41 (-0.33 to 1.16)	NW > FW
AverageA COP_X_ closed eyes	0.75 (-0.02 to 1.51)	FW > NW
AverageA COP_X_ open eyes	0.69 (-0.07 to 1.45)	FW > NW
AverageA COP_Y_ closed eyes	0.20 (-0.54 to 0.94)	NW > FW
AverageA COP_Y_ open eyes	0.12 (-0.62 to 0.86)	NW > FW
AverageSpeed COP_X_ closed eyes	0.22 (-0.52 to 0.96)	FW > NW
AverageSpeed COP_X_ open eyes	0.03 (-0.71 to 0.77)	FW > NW
AverageSpeed COP_Y_ closed eyes	0.06 (-0.68 to 0.80)	FW > NW
AverageSpeed COP_Y_ open eyes	0.13 (-0.61 to 0.87)	FW > NW
AverageSpeed_TOTAL_ closed eyes	0.13 (-0.61 to 0.87)	FW > NW
AverageSpeed_TOTAL_ open eyes	0.09 (-0.65 to 0.83)	FW > NW

## Discussion

The hypothesis of our study was rejected because eight weeks of NW and FW training promoted similar improvements in SWS, LRI, quality of life, static balance, and dynamic stability parameters. There were some differences between groups that we will report below. We highlight that the positive changes in SWS, LRI, quality of life, static balance and dynamic stability took place in a relatively short period (eight weeks). Also, the participants had a 90% adherence, thus demonstrating the effectiveness of the training program.

Our primary outcome, the SWS of both groups increased across time (FW group was 3.50 km.h^-1^ in pre-training to 4.00 km.h^-1^ in post-training; NW group was 4.00 km.h^-1^ in pre-training to 4.29 km.h^-1^ in post-training, Fig 4). These findings agree with Monteiro et al. [28] in relation to the increment of SWS in the factor time in FW and NW groups after six weeks of training and also regarding the higher walking speed values reported in NW group. However, it disagrees regarding the improvements related to the group effect with our study, probably because the elderly patients with Parkinson Disease are more sensitive to the use of poles, and the adaptations produced by NW in this population are also related to neural factors. However, our sedentary elderly participants reported some others improvements in physical condition. There was an increase in muscle strength of upper limbs and decrease of muscular pain. After the intervention period, both groups changed the training, so those who performed NW training during the intervention started to walk without poles, and the individuals from the FW group started walking with poles. And, afterward this training change, all participants reported their preference in walking with poles. Two main reasons may explain these positive perceptions stated by the individuals of the study: i) previous studies have shown a mismatch between the heart rate and effort perception using poles, indicating, in other words, that senior individuals using poles perform their training sessions at determined physiologic target (for example, percentage of maximal heart rate) with lower ratings of perceived exertion [29,30]. A reasonable supposition is that the relatively lower ratings of perceived exertion using poles in comparison to free walking may be associated with the positive observations relative to NW. ii) the superior stability derived from the using poles seems to be particularly interesting for the old people that have the postural control impaired, therefore, decreasing the risk of falls [30]. The perception of safety using poles during walking is a well-known characteristic of NW [5].

Similarly, an increase in walking speed of untrained older people was reported by Figueiredo et al. [5], being NW 106% more efficient than walking without poles in increasing walking speed of elderly people. Nevertheless, it should be noted that this walking speed was assessed through a 6-minute walk test (performed on track). Also, the participants of the present study were asked to the increase of the treadmill (0.5 km.h^-1^) for the determination of SWS and after declaring their SWS their speed was recorded and there were no changes or increases in speed as occurs in field tests. Yet, it is worth noticing that our results are in line with the findings of Takeshima et al. [4], who also did not find a difference in walking speed of sedentary elderly people after 12 weeks of NW training (50–70 minutes per day, three times a week) when compared to FW. Alike, the authors indicated that NW confers additional benefits in muscular strength when compared to walking without poles, also promoting improvements in aerobic capacity, muscle strength and in other components of functional fitness.

The LRI represents an integrative mechanism that minimizes muscular work and metabolic cost of walking. In the present study, the significant increase of LRI for both groups, with higher values for the NW group is associated with the increase in SWS in response to the use of poles. It follows a higher stride length and, therefore, reducing the total mechanical work by a better optimization of the center of mass energy exchanges [31].

Similarly, to SWS, the LRI presented significant increments for both groups. In a systematic review with meta-analysis [32], was established SWS normative values according to the mean age of people free of diseases between 20 and 99 years, grouped by decades. The results of the meta-analysis for the age group between 60–69 years (n = 941), considering the mean between the values for men and women, indicate the value of 4.64 km.h^-1^ as normative for this age group of healthy elderly, and our results are agree whit these study. Therefore, the improvements found with training in both groups approximated the values of SWS with the values considered normative for the age group analysed. The increase of SWS has a critical relevance in older people and is related to a decrease of 58% and 17% in relative and absolute risks of death, respectively. Interestingly, these benefits persist for eight years [33].

By analyzing our LRI results, there were significant improvements in time factor (*p* = 0.013) for both groups (FW group was from 68.08% to 77.85%; and NW group was from 76.73% to 82.26% from pre to post-training, respectively). The LRI was applied previously in individuals with Parkinson’s disease showing more significant improvements to NW group in comparison to FW group, thus showing a higher sensitivity to this particular population to training with poles, due to an improvement in coordination, balance, and functional mobility [34].

Additionally, we highlight that the LRI is also useful to identify changes in walking economy [19,20,28,35] indirectly. In other words, progressively during the locomotor training programs, the participants walk faster, and LRI shows how close this individual is to his/her theoretical optimal walking speed (in which less metabolic energy is consumed by distance travelled). Recently, the use of LRI was proposed as a new proposal to assess walking functionality, being extremely promising and indicating its application in studies that examine the effects of therapies on gait functionality in degenerative diseases [19]. We still point out that considering degenerative aspects related to the advanced aging in this study, LRI also has a preventive and motivational characteristic in physical conditioning of the elderly.

Moreover, we confirm that NW promotes gains at cardiovascular fitness, in upper limbs muscle strength and functional fitness [3,4,9,10]. These gains are important also in cognitive functions when NW training is performed along with vitamin D supplementation in women [36–38]. These outcomes, together with our findings, suggest the NW as an advantageous training method for the improvement of the physical condition of healthy elderly people. Lastly, we also highlight that its use could be applied to other populations (frail elderly, COPD patients, obese, among others).

Regarding the quality of life, we identified improvements in the psychological and social participation domains. These results corroborate other previous studies which identified exercise benefits in these domains in the elderly and other populations in clinical conditions [39–41]. The improvements occurred independently of the type of walking training. Several factors can explain the improvement of quality of life in psychological domain, like improvement in cognition, in self-reported functions and a decrease in depressive symptoms [42,43]. Our results corroborate with another study that applied NW training in frail elderly, older than 70 years (three weekly sessions with 60 minutes in duration, for 12 weeks). They compared NW against general exercises reported and there was a significant decrease in depression levels after intervention in NW group [6].

The results showed that both training interventions also were effective in improving the static balance of elderly people. This improvement was found by the reduction of COP average speed during the test in bipedal support, in both situations (open and closed eyes). However, the outcomes of COP amplitude did not show significant modifications after interventions. As expected, the results demonstrated higher values in the assessments open eyes in comparison to closed eyes condition. The decrease in COP’s speed values indicate a higher postural stability in these situations and corroborates with another study [44].

Thereby, the reduction in average speed of COP (COP_X_, COP_Y_ e COP_Total_) observed after the period training, indicated that both interventions were effective in producing the enhancement in postural control of elderly people. These findings corroborate other studies that also observed improvements in the static balance of older people after an NW intervention [6,45] or similar effects between NW and FW in this outcome [4,5]. However, these authors assessed static balance through time in unipodal support [6], functional reach test [4,45] and Berg’s scale [5]. Besides, no studies were found in the literature that used COP speed as the evaluation parameter of static balance, which hinders an in-depth analysis of this outcome with the NW training. One study [4] assessed the static balance in four static situations (firm and foam surface with eyes open and closed) in the elderly, but also they did not observe a decrease in static balance speed after 12 weeks of NW and FW training.

Conversely, in our study, the outcomes related to displacement amplitude of COP (Maximum Amplitude of COP_X_ and COP_Y_, Average Amplitude COP_X_, and COP_Y_) did not show differences after the training period. This result is contrary to Kocur et al. [45], who observed a reduction in COP maximal amplitude during functional tests after 12 weeks of NW training. They verified an increase in the distance reached in both tests after the intervention, indicating an improvement in postural sway. Still, we affirm that the improvement of static balance leads to the elderly an enhancement in postural stability, being able to reduce the risk of falls. Additionally, we recommend that older people engage in training programs regularly form, and also we confirm that both proposals are adequate and effective in improving the static balance of this population, in line with previous findings of walking with poles in elderly people [8] and in people with degenerative disorders [34].

The gait variability parameters were not influenced by time (training) and group (FW versus NW) factors. The group of healthy elderly, in walking usual speeds (3 and 4 km.h^-1^), showed values similar to the present study. Also, Oliveira et al. [16] evaluated the CoV of healthy elderly walking and found _values_ similar to our study at the speed 4 km.h^-1^, being 3.50 and 4.30% for CoV_CT_ and CoV_ST._, respectively. CoV_CT_ was 2.63 in pre- to 2.38% and from 2.34% in pre- to 2.15% in post-training for the FW and NW group, respectively. Moreover, the CoV_SL_ of [16] was by 2.30%, a value that also corroborates with the present study findings.

In our study, at the higher speeds, both groups were considered more stable and safer, due to the lower variability, representing a lower risk of falls, independently of the group [16, 30, 45, 46]. Thus, we corroborate one more finding of clinical relevance for training and rehabilitation of elderly people, related to dynamic stability of walking with and without poles.

## Conclusions

In conclusion, our hypothesis (additional benefits from NW in comparison with FW) was rejected. The improvements in SWS (primary outcome), LRI, quality of life, static balance and dynamic stability parameters in older people training NW were similar to that training FW.

## Study limitations

It should be noted that it was not possible to collect data from walking with poles on the treadmill. We preferred to take care of the physical integrity of the elderly. However, when collecting without poles, it was possible to evaluate the locomotion in conditions similar to their daily activities.

## Perspectives for future studies

We believe that the intervention with NW still can be widely explored, and more randomized clinical trials are needed to generate consensus in the scientific community and the health professionals about periodization, intensity, and volume of NW training to be applied, taking into account the purpose to improve physical conditioning. A randomized clinical trial longer is also interesting. Equally, we emphasize that there is still a lack of studies in elderly with associated comorbidities like COPD, intermittent claudication, cardiac diseases, obesity, and still the application of training and monitoring of physical conditioning of pregnant women along pregnancy.

## Supporting information

S1 Table General dataset. (XLSX)

S2 Table Effect sizes. (DOCX)

S1 File CONSORT checklist. (DOC)

S2 File Register in the clinical trials. (PDF)

S3 File Translated ethics committee. (DOCX)

S4 File Original ethics committee. (PDF)

S5 File TIDieR checklist. (DOCX)

S6 File Protocol study. (DOCX)

## Abbreviations

AM_AX_COP_X_: Amplitude Maximal COP_X_
AM_AX_COP_Y_: Amplitude Maximal COP_Y_
A_verage_ACOP_X_: Average Amplitude COP_X_
A_verage_ACOP_Y_: Average Amplitude COP_Y_
COP: center of pressure
CoV: coefficient of variation
CoV_CT_: coefficient of variation of contact time
CoV_SF_: coefficient of variation of stride frequency
CoV_SL_: coefficient of variation of stride length
CoV_ST_: coefficient of variation of swing time
FW: free walking or walking without poles
GEE: Generalized Estimating Equations
LRI: locomotor rehabilitation index
NW: Nordic walking or walking with poles
SWS: self-selected walking speed
