# Supplementary material for: Effects of Nordic walking training on quality of life, balance and functional mobility in elderly: A randomized clinical trial
Source: PLoS One. 2019 Jan 30;14(1):e0211472. doi: 10.1371/journal.pone.0211472 (PMC6353202; doi:10.1371/journal.pone.0211472)
Supplement: S2 Table — (DOCX) [file pone.0211472.s002.docx]

**Supplementary Material 2 –Table – Effect Sizes**

Different size effects analysis of quality of life test, static balance parameters, SWS and LRI can be observed on Table 1.

| **Table S1:** Effect size results of post-intervention comparisons of NW and FW groups. Values in mean and lower and upper limits of 95% Confidence Interval, for SWS, LRI, quality of life test and static balance parameters. | | |
| --- | --- | --- |
| **Variables:** | **NW vs FW**  **Mean (95% CI)** | **NW vs FW**  **Mean (95% CI)** |
| **SWS** | 0.42 (-0.31 to 1.16) |  |
| **LRI** |  | 0.36 (-0.39 to 1.10) |
| **- QoL Domains:** |  |  |
| BREEF – Physical | 0.50 (-0.28 to 1.29) |  |
| BREEF - Psychological | 0.25 (-0.53 to 1.02) |  |
| BREEF- Environment | 0.79 (-0.01 to 1.59) |  |
| BREEF – Social Relation | 0.90 (0.09 to 1.71) |  |
| BREEF – General QoL | 0.64 (-0.15 to 1.43) |  |
| OLD – Total | 0.05 (-0.72 to 0.82) |  |
| OLD – Social Participation |  | 0.02 (-0.75 to 0.79) |
| OLD – Sensory habilities | 0.28 (-0.49 to 1.06) |  |
| OLD – Past, present and future activities | 0.04 (-0.73 to 0.81) |  |
| OLD – Intimacy | 0.03 (-0.74 to 0.80) |  |
| OLD – Death and dying | 0.30 (-0.47 to 1.08) |  |
| OLD - Autonomy | 0.53 (-0.26 to 1.31) |  |
| **- Balance parameters**  **Static:** |  |  |
| AMax COP_X_ BL |  | 0.60 (-0.16 to 1.36) |
| AMax COP_X_ WB | 0.16 (-0.58 to 0.90) |  |
| AMax COP_Y_ BL | 0.14 (-0.60 to 0.88) |  |
| AMax COP_Y_ WB | 0.41 (-0.33 to 1.16) |  |
| AMean COP_X_ BL |  | 0.75 (-0.02 to 1.51) |
| AMean COP_X_ WB |  | 0.69 (-0.07 to 1.45) |
| AMean COP_Y_ BL | 0.20 (-0.54 to 0.94) |  |
| AMean COP_Y_ WB | 0.12 (-0.62 to 0.86) |  |
| MeanSpeed COP_X_ BL |  | 0.22 (-0.52 to 0.96) |
| MeanSpeed COP_X_ WB |  | 0.03 (-0.71 to 0.77) |
| MeanSpeed COP_Y_ BL |  | 0.06 (-0.68 to 0.80) |
| MeanSpeed COP_Y_ WB |  | 0.13 (-0.61 to 0.87) |
| MeanSpeed_TOTAL_ BL |  | 0.13 (-0.61 to 0.87) |
| MeanSpeed_TOTAL_ WB |  | 0.09 (-0.65 to 0.83) |

Effect sizes results for dynamic balance variables can be observed on Table 2.

| **Table 2:** Results in mean and lower and upper limits (95% CI) of effect size calculated for comparisons of post-intervention results of NW and FW groups in dynamic variability parameters. | | | | | |
| --- | --- | --- | --- | --- | --- |
|  | | | **Speeds** | | |
| **Variables** | **1 km.h^-1^**  **ES (95% CI)** | **2 km.h^-1^**  **ES (95% CI)** | **3 km.h^-1^**  **ES (95% CI)** | **4 km.h^-1^**  **ES (95% CI)** | **5 km.h^-1^**  **ES (95% CI)** |
| CoV_SL_ | 0.03 (-0.71 to 0.77) | 0.28 (-0.46 to 1.03) | 0.10 (-0.65 to 0.84) | 0.16 (-0.58 to 0.90) | 0.43 (-0.32 to 1.18) |
| CoV_SF_ | 0.05 (-0.69 to 0.79) | 0.30 (-0.45 to 1.04) | 0.06 (-0.68 to 0.80) | 0.16 (-0.58 to 0.90) | 0.45 (-0.30 to 1.20) |
| CoV_ST_ | 0.05 (-0.69 to 0.79) | 0.15 (-0.60 to 0.89) | 0.29 (-0.45 to 1.04) | 0.14 (-0.61 to 0.88) | 0.06 (-0.68 to 0.81) |
| CoV_CT_ | 0.31 (-0.44 to 1.05) | 0.54 (-0.22 to 1.29) | 0.29 (-0.46 to 1.06) | 0.24 (-0.51 to 0.98) | 0.20 (-0.54 to 0.94) |
