# Supplementary material for: Effects of Nordic walking training on quality of life, balance and functional mobility in elderly: A randomized clinical trial
Source: PLoS One. 2019 Jan 30;14(1):e0211472. doi: 10.1371/journal.pone.0211472 (PMC6353202; doi:10.1371/journal.pone.0211472)
Supplement: S2 File — (PDF) [file pone.0211472.s004.PDF]

ClinicalTrials.gov Protocol Registration and Results System (PRS) Receipt
Release Date: March 24, 2017

ClinicalTrials.gov ID: NCT03096964

Study Identification

Unique Protocol ID: LTartaruga,

Brief Title: Nordic Walking Training for Older People (Pole walking)

Official Title: Effects of Nordic Walking Training in Energetic, Mechanical and Neuromuscular Parameters of Elderly. A Randomized Clinical Trail

Secondary IDs:

Study Status

Record Verification: March 2017

Overall Status: Completed

Study Start: March 2014 []

Primary Completion: October 2014 [Actual]

Study Completion: December 2016 [Actual]

Sponsor/Collaborators

Sponsor: Federal University of Rio Grande do Sul

Responsible Party: Principal Investigator
Investigator: Leonardo A. Peyré-Tartaruga [ltartaruga,]
Official Title: Adjunct Professor
Affiliation: Federal University of Rio Grande do Sul

Collaborators:

Oversight

U.S. FDA-regulated Drug:

U.S. FDA-regulated Device:

IND/IDE Protocol: No

Human Subjects Review: Board Status: Approved
Approval Number: 878736
Board Name: Ethics committee of Federal University of Rio Grande do Sul
Board Affiliation: Federal University of Rio Grande do Sul
Phone: +55(51)3308-3738
Email: etica@propesq.ufrgs.br
Address:

110 Paulo da Gama Avenue - Room 317

Data Monitoring: No

Plan to Share IPD: No

FDA Regulated Intervention: No

Study Description

Brief Summary: Objective: to assess by means of a randomized clinical trial the effects of eight weeks of Nordic walking and free walking training on quality of life (QoL), static balance, dynamic variability, self-selected walking speed (SWS) and locomotor rehabilitation index (LRI), parameters of pendular mechanism [external (Wext), internal (Wint) and total mechanical work (Wtot), Recovery (R), Cost of Transport (C)], Electromyographic parameters (Average signal and co-contraction of the Anterior deltoid (AD), triceps brachii (TB), vastus lateralis (VL), biceps femoris (BF), anterior tibial (AT) and medial gastrocnemius (MG) muscles, heart rate of exercise (HRexercise) - and the rate of perceived exertion (RPE), in sedentary elderly.

Detailed Description: Introduction: Considering the increase in the elderly population and their life expectancy, together with the expansion of Nordic walking (NW) interventions as a training method for the elderly, there is a need of preventive studies of RCTs and with methodological good quality approach to enable physical education, health and rehabilitation professionals to make decisions regarding the type, the volume and the intensity of NW exercise in promoting the health of the sedentary elderly. Objectives: The aim of this study is to evaluate, through a randomized clinical trial, the effects of eight weeks of NW and FW training on quality of life (QoL), static balance, dynamic variability, self-selected treadmill walking speed (SSWSTreadmill), Locomotor rehabilitation (LRI) Parameters of Pendular Mechanism (external, internal and total mechanical work -Wext, Wint, Wtot, Recovery -R,Cost of Transport - C), Electromyographic parameters (Average signal and co-contraction of the AD, TB, VL, BF, AT and MG muscles), heart rate of exercise - HRexercise and the rate of perceived exertion (RPE), in sedentary elderly. of sedentary elderly. Experimental Design: Randomized Controlled Clinical Trial (RCT). Study Site: School of Physical Education, Physiotherapy and Dance (ESEFID), Federal University of Rio Grande do Sul Porto Alegre, Rio Grande do Sul, Brazil. Methods: The sample comprised 33 sedentary participants randomly divided into two groups (Nordic walking group and Free walking group who will perform training for 8 weeks.

Conditions

Conditions: Sedentary Lifestyle
Age Problems

Keywords: Elderly training
Pole walking
Locomotor Rehabilitation Index
Self-selected speed of walking
Recovery

Study Design

Study Type: Interventional

Primary Purpose: Treatment

Study Phase: N/A

Interventional Study Model:	Parallel Assignment
Number of Arms:	2
Masking:	Outcomes Assessor
Allocation:	Randomized
Enrollment:	32 [Actual]

Arms and Interventions

Arms	Assigned Interventions
Experimental: Nordic walking<br>Experimental: Nordic walking Training The total period of training was composed by 8-week of walking with poles, three sessions per week. The cycles were divided into eight microcycles composed by three training sessions. Each training session took 60 min. Nordic walking aerobics training were used during the training period. These exercises were performed alternating volume and intensity. Exercise intensities were controlled using the cardiac monitor, and the zone was since 70% to 105% of the heart rate at the second ventilatory threshold. And volume was controlled using the time of the session.	Nordic walking training<br>The training period begin for both groups, will be comprised of eight weeks of duration, with frequency of three weekly sessions. Volume (session duration in minutes) and intensity (percentage of the heart rate at second threshold) were the same for both groups, only differing in the use (NW) or not of the poles (FW). Each session will have of the Initial Part or warming-up, the Main Part and Final Part or cooling-down.
Experimental: Free Walking<br>Experimental: Free walking Training The total period of training was composed by a 8-week cycles of walking without poles, with three sessions per week. The cycles were divided into eight microcycles composed by three training sessions. Each training session took 60 min. Free walking aerobics training were used during the training period. These exercises were performed alternating volume and intensity. Exercise intensities were controlled using the cardiac monitor, and the zone was since 70% to 105% of the heart rate at the second ventilatory threshold. And volume was controlled using the time of the session.	Free walking training<br>The training period begin for both groups, will be comprised of eight weeks of duration, with frequency of three weekly sessions. Volume (session duration in minutes) and intensity (percentage of the heart rate at second threshold) were the same for both groups, only differing in the use (NW) or not of the poles (FW). Each session will have of the Initial Part or warming-up, the Main Part and Final Part or cooling-down.

Outcome Measures

Primary Outcome Measure:

1. Self-selected walking speed - SSWS
   This outcome will be measure through sub-maximal test of walking treadmill, using the portable gas analyser Equipment.

   [Time Frame: Change from baseline SSWS at 8 weeks]

Secondary Outcome Measure:

2. Quality of life (QoL)
   The quality of life will be estimated using the World Health Organization Quality of Life Project for elderly people (WHOQOL) instrument.

   [Time Frame: Change from baseline QoL at 8 weeks]
3. Locomotor rehabilitation index (LRI)
   This outcome will be measure through of the measure on sub-maximal test of walking treadmill, using the three-dimensional motion analysis system (VICON).

[Time Frame: Change from baseline LRI at 8 weeks]

4. Oxygen consumption (VO2)
   This outcome will be measure through of the of the walking test on treadmill and too using the portable gas analyzer equipment (units in ml/kg/min).

   [Time Frame: from baseline to 8 weeks]

5. Cost of transport (C)
   This outcome will be measured through of the walking test on a treadmill will be collected the oxigem consumption using the portable gas analyzer equipment (measure unit in ml.O2.km).

   [Time Frame: from baseline to 8 weeks]

6. Optimal Walking Speed (OPT)
   This outcome will be measure through of the registered image movement analysis using the three-dimensional motion analysis system (VICON) of the walking test on treadmill (unit in km/h)

   [Time Frame: from baseline to 8 weeks]

7. Oxygen Consumption at anaerobic threshold (VO2VT2)
   This outcome will be measure through maximal progressive treadmill test, using the portable gas analyser VO2000 equipment.

   [Time Frame: from baseline to 8 weeks]

8. Peak Oxygen Consumption (VO2peak)
   This outcome will be measure through maximal progressive treadmill test, using the portable gas analyser VO2000 equipment.

   [Time Frame: from baseline to 8 weeks]

9. Heart Rate at anaerobic threshold (HRVT2)
   This outcome will be measure through maximal progressive treadmill test, using a heart rate monitor (POLAR, FT1).

   [Time Frame: from baseline to 8 weeks]

10. Rest Heart Rate (HRrest)
    This outcome will be measure using a heart rate monitor (POLAR, FT1). The participants will remain seated in a quiet and controlled temperature room during 15 minutes prior to measurement of the heart rate.

    [Time Frame: from baseline to 8 weeks]

11. Exercise Heart Rate (HRexercise)
    This outcome will be measure using a heart rate monitor (POLAR, FT1) measurement of the heart rate of exercise during the walking test on treadmill at different speed.

    [Time Frame: from baseline to 8 weeks]

12. Rest Arterial Systolic Blood Pressure (SBPrest)
    This outcome will be measured through blood pressure ambulatory monitor (Meditech). The participants will remain seated in a quiet and controlled temperature room during 15 minutes prior to measurement of the arterial systolic blood pressure.

    [Time Frame: from baseline to 8 weeks]

13. Rest Arterial Diastolic Blood Pressure (DBPrest)
    This outcome will be measured through blood pressure ambulatory monitor (Meditech, ABPM-04 equipment). The participants will remain seated in a quiet and controlled temperature room during 15 minutes prior to measurement of the arterial systolic blood pressure.

    [Time Frame: Change from baseline DBPrest at 8 weeks]

14. Parameters of Static balance (Amplitude ans Speed of Center of pressure in X, Y and Z axis)
    This outcome will be measure through of Static Balance in Force Platform (AMTI)

    [Time Frame: Change from baseline Parameters of Static Balance at 8 weeks]

15. Parameters of Dynamic variability (coefficient of variation (CoV) of Stride length (SL), stride frequency (SF), swing time (ST), and contact time (CT)

This outcome will be measure through of the variability on sub-maximal test of walking treadmill, using the three-dimensional motion analysis system (VICON).

[Time Frame: Change from baseline Parameters of Dynamic Balance at 8 weeks]

16. Spatial Temporal Parameters (stride frequency, stride length, swing time and contact time)
This outcome will be measure through of the registered image movement analysis using the three-dimensional motion analysis system (VICON) of the walking test on treadmill at different speed of walking (1,2,3,4 and 5 km/h), 5 minutes at each speed.

[Time Frame: Change from baseline Spatial Temporal parameters at 8 weeks]

17. Parameters of Pendular Mechanism (external, internal and total mechanical work, Wext, Wint and Wtot, respectively, Recovery).
This outcome will be measure through of the registered image movement analysis using the three-dimensional motion analysis system (VICON) of the walking test on treadmill at different speed of walking (1,2,3,4 and 5 km/h), 5 minutes at each speed.

[Time Frame: Change from baseline Parameters of Mechanical Work at 8 weeks]

18. Other Parameters of Pendular Mechanism (Percentage of congruity (%Cong), instantaneous pendular transduction and Recovery of step, shift phase (PhaseShift).
This outcome will be measure through of the registered image movement analysis using the three-dimensional motion analysis system (VICON) of the walking test on treadmill at different speed of walking (1,2,3,4 and 5 km/h), 5 minutes at each speed.

[Time Frame: Change from baseline other Parameters of Pendular Mechanism at 8 weeks]

19. Movement Body Center of Mass (BCoM) on X, Y, Z axis
This outcome will be measure through of the registered image movement analysis using the three-dimensional motion analysis system (VICON) of the walking test on treadmill at different speed of walking (1,2,3,4 and 5 km/h), 5 minutes at each speed.

[Time Frame: Change from baseline of BCoM at 8 weeks]

20. Maximal voluntary contractions (MVC) of the muscles: anterior deltoid (AD), triceps brachii (TB), vastus lateralis (VL), biceps femoris (BF), anterior tibial (AT) and medial gastrocnemius (MG)
This outcome will be measured through Measuring the electromyographic activation during treadmill walking tests using the Miotool electromyograph

[Time Frame: Change from baseline MVC at 8 weeks]

21. Electromyographic Parameters (mean amplitude of the signal and co-contraction) of the muscles: anterior deltoid (AD), triceps brachii (TB), vastus lateralis (VL), biceps femoris (BF), anterior tibial (AT) and medial gastrocnemius (MG)
This outcome will be measured through Measuring the electromyographic activation during treadmill walking tests using an electromyograph

[Time Frame: Change from baseline Electromyographic parameters at 8 weeks]

Eligibility

Minimum Age: 60 Years

Maximum Age: 80 Years

Sex: All

Gender Based:

Accepts Healthy Volunteers: Yes

Criteria: Inclusion Criteria:

- Sedentary elderly people
- Aged between 60 and 80

Exclusion Criteria:

- Smoking
- Show chronic pain or presence of migraine or nausea in daily life;
- History of labyrinthitis

Contacts/Locations

Central Contact Person: Leonardo A Peyré-Tartaruga, PhD.
Telephone: +555133085820
Email: leonardo.tartaruga@ufrgs.br

Central Contact Backup: Natalia A Gomeñuka, Phd Student
Telephone: +555333121385
Email: natyg2412@gmail.com

Study Officials: Leonardo A Peyré-Tartaruga, PhD
Study Principal Investigator
PhD student of the Federal University of Rio Grande do Sul- Brasil

Locations: Brazil
Federal University of Rio Grande do Sul
Porto Alegre, Rio Grande do Sul, Brazil, 90690-200

References

Citations:

Links:

Study Data/Documents:

U.S. National Library of Medicine | U.S. National Institutes of Health | U.S. Department of Health & Human Services
