# Supplementary material for: Effects of Nordic walking training on quality of life, balance and functional mobility in elderly: A randomized clinical trial
Source: PLoS One. 2019 Jan 30;14(1):e0211472. doi: 10.1371/journal.pone.0211472 (PMC6353202; doi:10.1371/journal.pone.0211472)
Supplement: S3 File — (DOCX) [file pone.0211472.s005.docx]

**Supplementary Material 3 – Translated ethics committee.**

**FEDERAL UNIVERSITY OF RIO GRANDE DO SUL / RESEARCH SECTOR**

**EVALUATION CONSUBSTANCED OF THE ETHICS COMMITTEE**

**DATA OF THE RESEARCH PROJECT**

**Research Title: ACUTE AND CHRONIC EFFECTS OF A NORMAL AND NORDIC WALKING TRAINING PROGRAM IN THE MECHANICAL, ENERGETIC AND NEUROMUSCULAR PARAMETERS AND THE RATE OF PERCEIVED EFFORT IN SEDENTARY ELDERLY.**

**Researcher: Leonardo Alexandre Peyré Tartaruga**

**Thematic Area:**

**Version: 2**

**CAAE: 33784014.7.0000.5347**

**Institution: FEDERAL UNIVERSITY OF RIO GRANDE DO SUL**

**Main Sponsor: School of Physical Education,**

**DATA OF THE EVALUATION –**

**Report No: 878,736**

**Date of the Report: 09/18/2014**

**Project presentation:**

**This is a doctoral project of the student Natalia Andrea Gomeñuka, together with the PPG in Sciences of the Human Movement of UFRGS, under the supervision of Prof. Leonardo Tartaruga. The project is centered on the evaluation of a normal and Nordic walking program for the sedentary elderly. The Nordic walk is the walking movement carried out with the help of specific poles for this activity. Among its main characteristics are the increase in heart rate, oxygen consumption, muscle activation, and speed of progression, however, there is no change in rate of the perceived exertion when compared to normal walking.**

**Objective:**

**To analyze the acute and chronic effects of a training program of normal walking and Nordic walking in the mechanical, energetic, neuromuscular parameters and the rate of perceived exertion in the sedentary elderly.**

**Risk and Benefit Assessment:**

**Properly described in both the project and the Brazil Platform.**

**Address: Av. Paulo Gama, 110 - Room 317 of the Building Annex 1 of the Rectory - Campus Centro**

**Neighborhood: Farroupilha, RS County: Telephone: (51) 3308-3738**

**CEP: 90.040-060Fax: (51) 3308-4085 E-mail: etica@propesq.ufrgs.br**

**PORTO ALEGRE**

**Page 1 of 2**

**FEDERAL UNIVERSITY OF RIO GRANDE DO SUL / RESEARCH PRÓ- REITORIA -**

**Continuation of Report: 878,736**

**Comments and Research Considerations:**

**Research with merit and properly presented.**

**Considerations Regarding Mandatory Filing Terms:**

**The mandatory terms are present and adequate (the TCLE has been restructured and in the current version presents coherent wording that can be understood by the research participants). Poster template for invitation to participants has been presented and is appropriate. The sample size is appropriately justified in the design body.**

**Recommendations:**

**Appropriate design.**

**Conclusions or Pending and List of Inadequacies:**

**It is suggested that the project be approved.**

**Status of Evaluation:**

**Approved**

**Needs Assessment of CONEP:No**

**Final Considerations at CEP's discretion:**

**Approved.**

**PORTO ALEGRE, November 20, 2014**

**Signed by:**

**MARIA DA GRAÇA CORSO DA MOTTA (Coordinator)**
