# Supplementary material for: Effects of Nordic walking training on quality of life, balance and functional mobility in elderly: A randomized clinical trial
Source: PLoS One. 2019 Jan 30;14(1):e0211472. doi: 10.1371/journal.pone.0211472 (PMC6353202; doi:10.1371/journal.pone.0211472)
Supplement: S4 File — (PDF) [file pone.0211472.s006.pdf]

PARECER CONSUBSTANCIADO DO CEP

DADOS DO PROJETO DE PESQUISA

Título da Pesquisa: EFEITOS AGUDOS E CRÔNICOS DE UM PROGRAMA DE TREINAMENTO DE CAMINHADA NORMAL E NÓRDICA NOS PARÂMETROS MECÂNICOS, ENERGÉTICOS E NEUROMUSCULARES E NA SENSAÇÃO SUBJETIVA DE ESFORÇO DE IDOSOS SEDENTÁRIOS.

Pesquisador: Leonardo Alexandre Peyré Tartaruga

Área Temática:

Versão: 2

CAAE: 33784014.7.0000.5347

Instituição Proponente: UNIVERSIDADE FEDERAL DO RIO GRANDE DO SUL

Patrocinador Principal: Escola de Educação Física da Universidade do Rio Grande do Sul

DADOS DO PARECER

Número do Parecer: 878.736

Data da Relatoria: 18/09/2014

Apresentação do Projeto:

Trata-se de projeto de doutoramento da aluna Natalia Andrea Gomeñuka junto ao PPG em Ciências do movimento Humano da UFRGS, sob a supervisão do Prof. Leonardo Tartaruga. O projeto está centrado na avaliação de um programa de caminhada normal e nórdica destinado a idosos sedentários. A caminhada nórdica é o movimento de caminhada realizado com o auxilio de bastões específicos para esta atividade. Entre suas principais características se destacam o aumento da frequência cardíaca, do consumo de oxigênio, da ativação muscular, e da velocidade de progressão, porém, não há alteração no índice de esforço percebido quando comparado à caminhada normal.

Objetivo da Pesquisa:

Analisar os efeitos agudos e crônicos de um programa de treinamento de caminhada normal e caminhada nórdica nos parâmetros mecânicos, energéticos, neuromusculares e na sensação subjetiva de esforço de idosos sedentários.

Avaliação dos Riscos e Benefícios:

Adequadamente descritos tanto no projeto quanto na Plataforma Brasil.

Comentários e Considerações sobre a Pesquisa:

Pesquisa com mérito e adequadamente apresentada.

Considerações sobre os Termos de apresentação obrigatória:

Os termos obrigatórios estão presentes e adequados (o TCLE foi reestruturado e na versão atual apresenta redação coerente e passível de ser compreendida pelos participantes da pesquisa).

Modelo do cartaz de convite aos participantes foi apresentado e está adequado.

O tamanho da amostra está adequadamente justificado no corpo do projeto.

Recomendações:

Projeto adequado.

Conclusões ou Pendências e Lista de Inadequações:

Sugere-se a aprovação do projeto.

Situação do Parecer:

Aprovado

Necessita Apreciação da CONEP:

Não

Considerações Finais a critério do CEP:

Aprovado.

PORTO ALEGRE, 20 de Novembro de 2014

Assinado por:
MARIA DA GRAÇA CORSO DA MOTTA
(Coordenador)

Endereço: Av. Paulo Gama, 110 - Sala 317 do Prédio Anexo 1 da Reitoria - Campus Centro
Bairro: Farroupilha **CEP:** 90.040-060
UF: RS **Município:** PORTO ALEGRE
Telefone: (51)3308-3738 **Fax:** (51)3308-4085 **E-mail:** etica@propesq.ufrgs.br
