# Supplementary material for: Effects of Nordic walking training on quality of life, balance and functional mobility in elderly: A randomized clinical trial
Source: PLoS One. 2019 Jan 30;14(1):e0211472. doi: 10.1371/journal.pone.0211472 (PMC6353202; doi:10.1371/journal.pone.0211472)
Supplement: S6 File — (DOCX) [file pone.0211472.s008.docx]

**Supplementary material 6 - Study Protocol**

The article entitled:

"EFFECTS OF NORDIC WALKING TRAINING ON QUALITY OF LIFE, BALANCE AND FUNCTIONAL MOBILITY IN ELDERLY: A RANDOMIZED CLINICAL TRIAL" corresponds to the second article of this thesis (study B of the abstract thesis), which is part of a larger study carried out in the doctoral thesis entitled:

**Title PhD Thesis:**

EFFECTS OF A NORDIC WALKING TRAINING PROGRAM IN THE MECHANICAL, ENERGETIC AND NEUROMUSCULAR PARAMETERS, QUALITY OF LIFE AND THE RATE OF PERCEIVED EXERTION OF SEDENTARY ELDERLY. A CONTROLLED RANDOMIZED CLINICAL TRIAL

# ABSTRACT THESIS

**Introduction:** Considering the rapid increase in the elderly population and their life expectancy, together with the expansion of Nordic walking (NW) interventions as a training method for the elderly, there is a need for experimental studies (randomized controlled trials, ECRs) of preventive character and of good methodological quality that allow the professionals of physical education, health and rehabilitation to make decisions regarding the type, volume and intensity of NW exercise in the health promotion of sedentary elderly. **Objectives:** This study aimed (**study A**) to determine, through a systematic review of randomized clinical trials, whether systematic training with NW, compared to free walking (FW), has beneficial effects on components of the functional capacity of sedentary elderly, also (**study B**) evaluate the effects of eight weeks of NW and FW training on quality of life (QoL), static balance, dynamic variability, self-selected walking speed on treadmill (SSWS) and Locomotor rehabilitation index (LRI) of sedentary elderly, and also evaluate (**study C**) the effects of eight weeks of NW and FW training on mechanical work (external, internal and total mechanical work -W_ext_,W_int_, W_tot_), pendular mechanism (*R*), Cost of transport (C), exercise heart rate (HR_exercise_), rate of perceived exertion (RPE), and electromyographic parameters (mean signal amplitude and co-contraction) of muscles: Anterior Deltoid (AD), Triceps Brachii (TB), Vastus Lateralis (VL), Femoral Biceps (BF), Anterior Tibialis (TA) and Medial Gastrocnemius (MG) of sedentary elderly. **Experimental Design:** Controlled Clinical Trial (ECR). **Research Location**: School of Physical Education, Physiotherapy and Dance (ESEFID), Federal University of Rio Grande do Sul, Porto Alegre, Rio Grande do Sul, Brazil. **Methods**: The sample comprised 33 sedentary participants randomly divided into two groups (NW group, n =16, age: 64.6 ± 4.1 years old, weight: 81.5 ± 10.7 kg and height: 166.3 ± 7.5 cm; and FW group, n =16, age: 68.6 ± 3.9 years, mass: 74.6 ± 14.5 kg and height: 161.6 ± 10.3 cm), performed NW and FW training for 8 weeks. **Outcomes of Study A:** Parameters of functional fitness of the elderly (upper limb strength and endurance, lower limb strength and endurance, mobility, lumbar spine and posterior hip muscle flexibility, the general amplitude of the shoulder joint, aerobic endurance, and self-selected speed). **Outcomes of study B:** Functional parameters (QoL, static and dynamic balance, SSWS and LRI). **Outcomes of Study C**: Mechanical parameters and pendular mechanism (W_ext_, W_int_, W_tot_, *R*, C, SSWS), electromyographic parameters (mean signal amplitude and Co-contraction of AD, TB, VL, BF, AT and MG muscles), HR_exercise_, and RPE of sedentary elderly. **Statistic Analysis:** Sample description data, at baseline, were compared by applying one-way ANOVA. The outcomes were analyzed using Generalized estimating equations (GEE), to compare the groups (NW and FW) and the moments (pre and post training) and the different speeds (1,2,3,4 and 5 km h^-1^). A Bonferroni post-hoc was used to identify the differences between effects and interactions. The data were presented in model-based adjusted means and were analyzed with the software Statistical Package for the Social Sciences (SPSS) v.22.0. A significance level of α = 0.05 was adopted. **Conclusion:** NW and FW training promotes improvements in functional fitness components; in the static balance and the dynamic variability of the elderly. However, some differences between the two interventions are related to the principle of training specificity and occur only in the components that received the most stimuli imposed by the walking technique with poles, especially in the upper limbs. Furthermore, the increase in SSWS on the treadmill combined with the use of LRI allows us to conclude that NW training has clinical relevance and is recommended as a means of improving physical conditioning and as a method of rehabilitation of sedentary elderly. This physical activity provides central adaptations with significant improvement in the functional mobility of the elderly. Thus, after aerobic resistance training with and without poles, the elderly increase the speed of locomotion in daily activities and with lower metabolic cost due to the central adaptations and improvement of the pendulum mechanism due to the greater proximity of the SSWS to the optimal speed of walking. There are still significant adaptations in muscle activation resulting from NW training in the elderly indicating a reduction in the levels of co-contraction of upper limbs in walking without poles, which directly affects the improvement of physical fitness and functional independence of the elderly. Also, we recommend NW as a safe and effective physical activity for this population when the periodization of training is performed with the volume and intensity strictly controlled.

**Keywords:** Walking with Poles, Locomotor Reabilitation Index, Self-selected walking speed, Recovery, Co-activation EMG.

**UNIVERSIDADE FEDERAL UNIVERSITY OF RIO GRANDE DO SUL. POST-GRADUATE PROGRAM ON HUMAN MOVEMENT SCIENCES**

**Author:** Natalia Andrea Gomeñuka

**Advisor:** Prof. Dr. Leonardo Alexandre Peyré-Tartaruga.

**Thesis:** Effects of Nordic walking on mechanical, energetic and neuromuscular parameters, quality of life and perceived exertion in sedentary elderly. A controlled randomized clinical trial.

Porto Alegre, December 2016.

**Trial Design**

**Sample**

All participants read and signed a free and informed consent form before starting their participation in the study (**number 878.736**). All evaluations and training sessions were conducted in the Physical Education, Physiotherapy and Dance School of the Federal University of Rio Grande do Sul (UFRGS) of Porto Alegre – Brazil. Thirty-two sedentary elderly people were randomized into two groups. The intervention group performed walking with poles training (NW) during eight weeks, and the control group accomplished walking without poles training (FW) during the same period. Volunteers were divided into NW group with 16 participants, age: 64.6±4.1 years old, body mass: 81.5±10.7 kg, and height: 166.3±7.5 cm; and FW group with 16 participants, age: 68.6±3.9 years old, body mass: 74.6±14.5kg and height: 161.6±10.3 cm. Sedentary elderly people, understood as those seniors who were at least six months without practicing structured or systematized physical activities, aged between 60 and 80, non-smoking, and who did not show chronic pain or presence of migraine or nausea in daily life, or history of labyrinthitis, and also that did not have factors that could impair the elderly to conclude the sessions and tests were included. Data of the seniors who did not have a minimum of 90% of training frequency were excluded from the analysis. Participants were allocated to the two groups by simple randomization, using a computer software that performs a binary random list. Allocation concealment was performed by a sequentially numbered list, in which a blinded evaluator indicated to each group each number (subject) corresponded. The researcher that conducted the allocation was an impartial evaluator who was not involved with the study, in order to maintain the confidentiality of the allocation and blinding of the study. Randomization and allocation processes were performed after the conclusion of the familiarization with Nordic walking technique and before starting the training.

**Study Design and Procedures**

This study was designed as a randomized controlled clinical trial in parallel, with allocation index of 1:1. There were no alterations in the groups after the beginning of the training. All subjects performed a period of one month of familiarization with Nordic walking technique (one weekly session of 45 minutes). After familiarization, subjects attended to the laboratory to undergo the pre-training period assessments.

On the first visit (day 1) anthropometric, quality of life (WHOQOL-OLD and WHOQOL-BREEF) and static balance in force platform assessments were performed. Anthropometric evaluations were conducted after the fulfilment of quality of life questionnaires. In pre- and post-training moments, these variables were collected by two trained researchers, who were impartial, not involved with the study and/or training and blinded to the allocation of the groups.

On the second visit (day 2), subjects performed an incremental maximal test in the treadmill. On the third visit (day 3) to the laboratory, subjects attended for the accomplishment of the evaluation of SSWS and walking in different submaximal speeds (1, 2, 3, 4 and 5 km.h^-1^) on the treadmill, where SSWS, LRI and dynamic balance in the pre-training moment. After the conclusion of these assessments, the subjects were randomized in NW and FW groups and performed eight weeks of training. After the training period, the subjects went back to the laboratory to undergo the same evaluations corresponding to post-training moment. During all intervention period, the subjects were instructed to continue with their usual prescribed medication.
